# Evaluation of a new pocket echoscopic device for focused cardiac ultrasonography in an emergency setting

**DOI:** 10.1186/cc11340

**Published:** 2012-05-14

**Authors:** Matthieu Biais, Cédric Carrié, François Delaunay, Nicolas Morel, Philippe Revel, Gérard Janvier

**Affiliations:** 1 Emergency Department, Hôpital Pellegrin, Centre Hospitalier Universitaire de Bordeaux, Place Amélie Raba Léon, F-33076 Bordeaux Cedex, France; 2 Université Bordeaux Segalen, Rue Léo Saignat, F-33076, Bordeaux Cedex, France

## Abstract

**Introduction:**

In the emergency setting, focused cardiac ultrasound has become a fundamental tool for diagnostic, initial emergency treatment and triage decisions. A new ultra-miniaturized pocket ultrasound device (PUD) may be suited to this specific setting. Therefore, we aimed to compare the diagnostic ability of an ultra-miniaturized ultrasound device (Vscan™, GE Healthcare, Wauwatosa, WI) and of a conventional high-quality echocardiography system (Vivid S5™, GE Healthcare) for a cardiac focused ultrasonography in patients admitted to the emergency department.

**Methods:**

During 4 months, patients admitted to our emergency department and requiring transthoracic echocardiography (TTE) were included in this single-center, prospective and observational study. Patients underwent TTE using a PUD and a conventional echocardiography system. Each examination was performed independently by a physician experienced in echocardiography, unaware of the results found by the alternative device. During the focused cardiac echocardiography, the following parameters were assessed: global cardiac systolic function, identification of ventricular enlargement or hypertrophy, assessment for pericardial effusion and estimation of the size and the respiratory changes of the inferior vena cava (IVC) diameter.

**Results:**

One hundred fifty-one (151) patients were analyzed. With the tested PUD, the image quality was sufficient to perform focused cardiac ultrasonography in all patients. Examination using PUD adequately qualified with a very good agreement global left ventricular systolic dysfunction (κ = 0.87; 95%CI: 0.76-0.97), severe right ventricular dilation (κ = 0.87; 95%CI: 0.71-1.00), inferior vena cava dilation (κ = 0.90; 95%CI: 0.80-1.00), respiratory-induced variations in inferior vena cava size in spontaneous breathing (κ = 0.84; 95%CI: 0.71-0.98), pericardial effusion (κ = 0.75; 95%CI: 0.55-0.95) and compressive pericardial effusion (κ = 1.00; 95%CI: 1.00-1.00).

**Conclusions:**

In an emergency setting, this new ultraportable echoscope (PUD) was reliable for the real-time detection of focused cardiac abnormalities.

## Introduction

The widespread use of emergency echocardiography provides valuable assistance in the management of acutely ill patients, allowing fast and accurate assessment of cardiac function and the ability to determine the cause of hemodynamic disorders [1-4]. Because standard echocardiographic equipment may be heavy and difficult to handle, hand-carried ultrasound (US) devices have been developed for bedside use, facilitating the growth of point-of-care ultrasonography [5]. When used by adequately trained intensivists, these devices have a well-demonstrated reliability and a widespread availability and rapid diagnostic capability, ideally suited for the emergency setting [6-8]. However, confusion between focused and comprehensive US among the many types of hand-carried US devices must be avoided [9-11]. Pocket devices are not able to perform a complete echocardiographic examination but can provide accurate diagnoses based on two-dimensional imaging for effective bedside screening. They can be used as an extension of the physical examination in various clinical settings, underlining the concept of personal echoscope. They have been shown to directly guide and alter clinical management and have a therapeutic impact equivalent to that of standard echocardiography [12-15].

Recently, a new generation of ultra-miniaturized US devices - the Vscan™ (GE Healthcare, Wauwatosa, WI, USA) - has been developed. Its true portability, ease of use, and relative low cost make this device adequately suited for routine use in strategies of focused US examination. At present, only a few studies evaluating its feasibility and clinical usefulness have been published [16-20]. However, none of them has compared its diagnostic accuracy with that of standard echocardiography in emergency settings. Thus, the main purpose of this study was to evaluate the recently available pocket US device (PUD) in comparison with standard transthoracic echocardiography (TTE) for focused cardiac ultrasonography in the emergency setting.

## Materials and methods

### Patients

During a 4-month period (from February to May 2011), all patients who were admitted to our emergency department and who required a TTE were included, unless two investigators were both unavailable. The following variables were collected at enrolment: demographic data, reason for admission (medical or surgical), medical history, cardio-respiratory status, Simplified Acute Physiology Score II (SAPS II), and indication for performing the US exam. Indications for TTE were left to the discretion of the attending physician but should be focused on a clinical problem or evaluation of patients with cardiac history (Table 1). This prospective, single-center, and observational study was approved by the local ethics committee (Comité de Protection des Personnes Sud-Ouest et Outre Mer III, Bordeaux, France; protocol DC 2011/07), which waived the need for informed consent. Patients or next of kin were orally informed of the goal and design of the study.

**Table 1:** Main characteristics of a population of 151 patients

Characteristics of population	Values
Age, years	55 ± 20
Females/Males, number	98/53
Simplified Acute Physiology Score II	35 ± 18
Invasive mechanical ventilation	66 (45)
Heart rate, beats per minute	81 ± 20
Mean arterial pressure, mm Hg	83 ± 19
Norepinephrine support	
Patients on norepinephrine support	38 (25)
Norepinephrine, μg/kg per minute	0.3 ± 0.3
Etiologies of emergency admission	
Surgical	108 (72)
Medical	43 (28)

Data are expressed as mean ± standard deviation or as number (percentage).

### Investigators and equipment

Each eligible patient was subsequently examined by two different echocardiography systems: the tested PUD (Vscan™; GE Healthcare) and a conventional echographic system (Vivid S5™; GE Healthcare). The new PUD, designed to be operated with one hand, consisted of a 135 × 73 × 28 mm unit connected to a 1.7 to 3.8 MHz phased array transducer (total weight of 390 g). Autonomy, battery fully charged, was 1 hour. The technical capabilities of the PUD allowed diagnosis based only on two-dimensional imaging and allowed possible adjustments of global gain and depth. Color flow mode was available, but there were no advanced features such as the M-mode or pulsed and continuous Doppler mode. Images could be frozen and stored for review.

Each examination was performed independently by two intensivists - MB and CC, who are experienced in echocardiography and have level II competence (as assessed by the European Association of Echocardiography) in general adult TTE - in random order within a 30-minute time frame. Both investigators were provided with medical history and clinical and paraclinical exams of the patient but were unaware of the results found by the alternative echocardiography device.

### Data acquisition

As recommended for focused cardiac examination in emergency patients [9], parameters recorded by using the PUD were the assessment of global cardiac systolic function, the identification of ventricular enlargement or hypertrophy, the assessment for a pericardial effusion, and the visual estimation of the size and respiratory changes of the inferior vena cava (IVC) diameter. Each clinical question was recorded by using a qualitative approach considered positive, negative, or undetermined (no response when the imaging quality or the device technology failed to provide a definite diagnosis). Only left ventricular ejection fraction (LVEF) was estimated by using a quantitative and visual approach.

For the purpose of this study, the systematic examination using conventional TTE was considered the reference diagnostic method to measure each variable, as recommended by the European Association of Echocardiography [21]. LVEF was calculated by the Simpson method unless it was impossible to obtain and then estimate the ejection fraction by visual approach. Hypertrophy was noticed when the thickness of the interventricular septum exceeded 13 mm, and dilation was noticed when the maximal end-diastolic antero-posterior diameter of the LV cavity was above 55 mm. Right ventricular (RV) dilation was defined by a diastolic ventricular ratio of greater than 0.6 when measured in the apical four-chamber view [22]. IVC diameter was measured proximally in the subcostal view between the right atrial junction and the superior hepatic vein, and a dilation corresponded to an end-expiratory diameter of greater than 23 mm [23]. IVC was considered collapsic if its diameter presented at least a 50% inspiratory decrease in patients with spontaneous ventilation [24]. Pericardial effusion, detected by an echo-free space, was considered compressive in the case of a collapse of the right cavities or respiratory variations of Doppler flow or both.

All echocardiography was interpreted online at bedside. For each US examination, the three standard transthoracic windows (parasternal, apical, and subcostal) were systematically screened. The examination was considered inconclusive when image quality was considered low, data were missing, or not accessible by the visual approach alone. In this case, the corresponding clinical question was not addressed. When all data were collected, duration of the study was measured for each method.

### Statistical analysis

Results are expressed as mean ± standard deviation or median (25% to 75% interquartile range) as appropriate. The results of the PUD and conventional TTE were compared in each patient. Results obtained with the conventional TTE were considered the reference. LVEFs obtained with the two devices were compared by using linear correlation and Bland and Altman analysis [25]. The ability of the PUD to discriminate the severity of the global LV systolic dysfunction (normal was greater than 50%, moderately depressed was 30% to 50%, and severely depressed was less than 30%) was tested by using Cohen's κ coefficient, and 95% confidence intervals (CIs) were calculated [26]. For each other clinical question, data were recorded in order to calculate the sensitivity, specificity, and positive and negative predictive values. The agreement between the clinical responses provided by the two devices was assessed by using Cohen's κ coefficient, and 95% CIs were calculated [26]. Kappa values of less than 0.2 were interpreted as slight, 0.21 to 0.4 as fair, 0.41 to 0.6 as moderate, 0.61 to 0.8 as substantial, and 0.81 to 1.00 as very good agreement [27]. The duration of the exam using the PUD or the conventional approach was compared by using the Wilcoxon test. A *P *value of less than 0.05 was considered statistically significant.

## Results

During the study period, 182 patients admitted to our department underwent a TTE. Among them, 31 patients were not enrolled in the study, because of the absence of at least one investigator. Finally, 151 patients were included. The main characteristics of these patients are shown in Table 1, and indications for TTE are shown in Table 2.

**Table 2:** Indications for transthoracic echocardiography in a population of 151 patients

Indications for transthoracic echocardiography	Patients, number (percentage)
Systematic or preoperative evaluation in patient with cardiovascular history	49 (32)
Hypotension, shock, or postcardiac arrest	38 (25)
Chest trauma exploration without respiratory or hemodynamic failure	34 (23)
Acute respiratory distress	20 (13)
Chest pain	7 (5)
Arterial embolism	3 (2)

**Figure 1:**
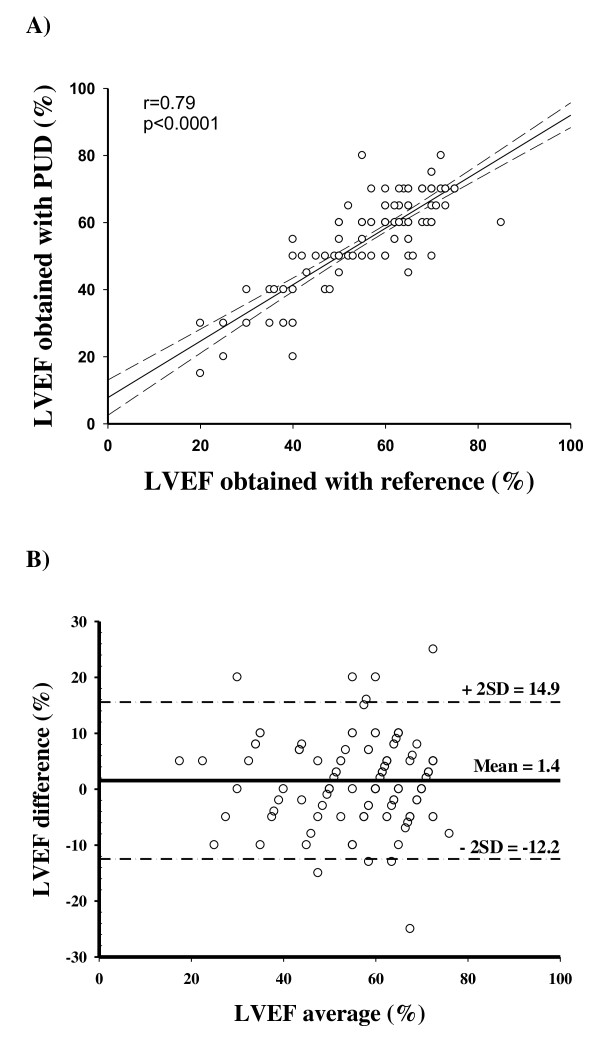
**Comparison of left ventricular ejection fractions (LVEFs) obtained by two devices**. LVEFs were compared by using **(a) **linear correlation and **(b) **Bland and Altman analysis. A conventional high-quality echocardiography system was considered the reference. PUD, pocket ultrasound device; SD, standard deviation.

The ability of the PUD to diagnose clinical problems is shown in Table 3, and sensibility, specificity, positive and negative predictive values, and κ coefficient are compared with those of a standardized examination. LVEFs obtained with PUD and with conventional echocardiography were not significantly different (58% ± 13% versus 59% ± 13%, respectively, *P *> 0.05) and were correlated (*r *= 0.79, 95% CI 0.72 to 0.84, *P *< 0.0001) (Figure 1). Comparison between LVEFs obtained with PUD and with conventional TTE showed a mean bias of 1.4% and limits of agreement of -12.2% to 14.9% (Figure 1). The intra-observer reproducibility percentages were 6.6% ± 3.3% using the standard TTE and 2.6% ± 4.2% using the PUD. The inter-observer reproducibility percentages were 6.9% ± 4.3% using standard TTE and 5.5% ± 3.9% using the PUD. Furthermore, examination using PUD adequately qualified the severity of the global LV systolic dysfunction (normal, moderately depressed, or severely depressed) in 134 patients (κ = 0.87, 95% CI 0.76 to 0.97). The presence (or absence) of LV dilation or hypertrophy was adequately classified by PUD, and there was substantial agreement (κ > 0.60). Compared with conventional TTE, PUD adequately identified RV dilation, RV dysfunction, pericardium effusion, and tamponade with good to excellent agreement (range of κ values was 0.63 to 1). Examination using PUD adequately identified the IVC size: empty (κ = 0.86, 95% CI 0.77 to 0.94) or dilated (κ = 0.90, 95% CI 0.80 to 1.00). Respiratory-induced variations in IVC size were well evaluated in spontaneous breathing (κ = 0.84, 95% CI 0.71 to 0.98).

**Table 3:** Ability of miniaturized echocardiographic system to diagnose clinical problems in a population of 151 patients

	Number of patients	Se, percentage (95% CI)	Sp, percentage (95% CI)	PPV, percentage (95% CI)	NPV, percentage (95% CI)	κ (95% CI)
Left ventricle						
Left ventricular ejection fraction < 50%	28	86 (69-94)	99 (96-100)	96 (80-99)	97 (93-99)	0.89 (0.79-0.98)
Left ventricular dilation	8	94 (61-99)	96 (91-98)	57 (32-79)	100 (97-100)	0.70 (0.48-0.93)
Left ventricular hypertrophy	26	77 (58-89)	97 (92-99)	83 (64-93)	95 (90-98)	0.76 (0.62-0.90)
Right ventricle						
Right ventricular dilation, cutoff of 0.6	21	59 (39-77)	98 (94-99)	87 (62-96)	93 (87-96)	0.66 (0.47-0.85)
Severe right ventricular dilation, cutoff of 1	12	92 (65-98)	99 (95-100)	85 (59-96)	99 (96-100)	0.87 (0.72-1)
Pericardum						
Pericardial effusion	11	91 (62-89)	96 (92-98)	67 (42-85)	99 (96-100)	0.75 (0.55-0.95)
Compressive pericardial effusion	2	100 (34-100)	100 (97-100)	100 (34-100)	100 (97-100)	1 (1-1)
Inferior vena cava (IVC)						
IVC dilation	20	85 (64-96)	100 (97-100)	97 (78-100)	98 (93-99)	0.90 (0.80-1.00)
Respiratory variations of IVC size in SB patients	61	97 (89-99)	87 (67-95)	95 (87-98)	91 (71-97)	0.84 (0.71-0.98)

κ, kappa coefficient; CI, confidence interval; NPV, negative predictive value; PPV, positive predictive value; SB, spontaneously breathing; Se, sensitivity; Sp, specificity.

Image quality was good enough to address all clinical questions estimated by visual approach alone without missing data. Compared with conventional TTE exam, the exam using PUD was significantly shorter: 420 seconds (300 to 600) versus 180 seconds (165 to 300), respectively (*P *< 0.0001).

## Discussion

Our study suggested that, in an emergency setting, the new PUD was reliable for the detection of focused cardiac abnormalities with a good agreement when compared with a last-generation conventional US device. Our results are in accordance with those of previous studies evaluating the diagnostic capabilities of this PUD in other clinical situations. In 100 cardiologic patients in a cardiological setting, Lafitte and colleagues [19] demonstrated that PUD showed good concordance of diagnostic capability with that of standard full-feature echocardiographic instruments. Prinz and Voigt [28], who studied 349 consecutive patients from an echocardiography lab, showed a very high image quality, excellent LVEF and LV dimension evaluations, and a good detection of pericardial effusion. In a perioperative setting, Frederiksen and colleagues [18] confirmed that PUD was well suited for performing a focus-assessed TTE in a surgery setting with a good image quality. More recently, Amiel and colleagues [16] demonstrated that PUD was able to assess LVEF with a good agreement in 94 critically ill patients.

In emergency or critical care settings, focused US allows findings to be directly correlated with the clinical situation, thereby offsetting the inherent limitations of the physical exam with real-time cardiovascular imaging [29]. Moreover, limited TTE not only provides new information on cardiac function but also may change the initial management of intensive care patients with a similar therapeutic impact thanks to the use of a portable US system compared with a standard US, despite its lower overall diagnostic capacity [13,15]. By increasing the number of clinical diagnoses, PUD could prove cost-effective by decreasing the number of medical errors, providing a more efficient real-time diagnosis, and reducing the use of unnecessary routine US examinations [17,30]. The capability and simplicity of this new PUD make it ideally suited for bedside use in emergency settings, providing time-sensitive assessment to assist physicians in the diagnosis of cardiovascular diseases in addition to the physical examination [31,32]. In fact, it embodies the concept of the visual stethoscope by Galderisi and colleagues [33], who demonstrated the relevant additional diagnostic power of pocket-size devices in addition to the physical examination. Thanks to a semi-quantitative approach, the device can be used as an echoscopic tool, differing from echocardiography in the lack of quantitative and dedicated measurement tools (that is, area and volume calculations), the absence of flow Doppler capacity, and the inability to provide an extensive report. Recently, a consensus statement by the American Society of Echocardiography/American College of Emergency Physicians emphasized the complementary role of focused cardiac US to that of a more comprehensive echocardiography [9]. The semi-quantitative evaluation of cardiac function by focused US cannot replace a more complete echocardiographic examination but, in some cases, may specify its indications without delaying the immediate management of severe cardiac dysfunction as suspected by the clinical examination.

The smallness of these devices does not preclude the need for full training. Ultrasonography is a user-dependent technology, and as usage spreads, clinicians' skills need to be ensured and the benefits of its appropriate use need to be defined. Most of the recommendations that define skills required in critical care or emergency US also insist on the necessary training required to perform an appropriate examination for a specific purpose [1,4,34]. Furthermore, to apply a systematic diagnostic and therapeutic protocol based on US, all emergency physicians have to be trained in echocardiography, and completing this training can be challenging.

The present study has several limitations. First, the way data were collected was different between the two techniques: we used quantitative measurements with classic US and qualitative assessment with PUD. Consequently, the difference in time consumption was probably explained by the realization of time-specific measures for the evaluation of diameters and ejection fractions with the reference device. Similarly, intra-observer variability assessment was biased by the visual approach, as illustrated by lower intra-observer but similar inter-observer variability. However, the systematic measures using the reference diagnostic method were necessary to confirm that a visual approach alone may be sufficient [35]. Second, we found that the image quality of PUD was sufficient for focused echocardiography, but this does not mean that image quality was similar between the two devices. Third, both PUD and classic TTE were performed by operators experienced in echocardiography and therefore sensitized to a visual assessment of semi-quantitative parameters. We cannot evaluate the relevance of this device if used by an examiner who received minimal training in US. Finally, we evaluated an ultra-miniaturized PUD for focused cardiac ultrasonography in emergency patients but did not assess the impact of this evaluation in terms of diagnostic strategy, treatment, or prognosis.

## Conclusions

We showed that, in an emergency setting, a new PUD is reliable for the detection of focused cardiac abnormalities with a good agreement when compared with a last-generation conventional US device. The former device cannot replace a more comprehensive echocardiographic evaluation but can trigger a more precise request without delaying the management of acutely ill patients. However, further methodologically rigorous studies are needed to assess patient-centered outcomes for point-of-care US.

## Key messages

• The image quality of the tested pocket ultrasound device was sufficient to perform focused cardiac ultrasonography in an emergency setting.

• This new pocket ultrasound device is reliable for the detection of focused cardiac abnormalities by a semi-quantitative visual analysis with good agreement.

• Our results should not be extrapolated to comprehensive echocardiography.

• The training necessary to achieve a sufficient level of competence remains to be determined.

## Abbreviations

CI: confidence interval; IVC: inferior vena cava; LV: left ventricular; LVEF: left ventricular ejection fraction; PUD: pocket ultrasound device; RV: right ventricular; TTE: transthoracic echocardiography; US: ultrasound.

## Competing interests

The authors declare that they have no competing interests.

## Authors' contributions

MB helped to conceive the study and design the trial, supervised the conduct of the trial and data collection, and helped to perform all echocardiography, to provide statistical advice on study design, to analyze the data, and to draft the manuscript. CC helped to conceive the study and design the trial, to perform all echocardiography, to provide statistical advice on study design, to analyze the data, and to draft the manuscript. GJ helped to conceive the study and design the trial. NM, FD, and PR helped to undertake recruitment of participating patients. All authors read and approved the final manuscript.

## References

[B1] American College of Emergency Physicians Emergency ultrasound guidelines Ann Emerg Med 2009 53 550 570 19303521 10.1016/j.annemergmed.2008.12.013

[B2] Beaulieu Y Bedside echocardiography in the assessment of the critically ill Crit Care Med 2007 35 S235 249 10.1097/01.CCM.0000260673.66681.AF 17446784

[B3] Nelson BP Melnick ER Li J Portable ultrasound for remote environments, Part I: Feasibility of field deployment J Emerg Med 2011 40 190 197 10.1016/j.jemermed.2009.09.006 20097500

[B4] Price S Via G Sloth E Guarracino F Breitkreutz R Catena E Talmor D Echocardiography practice, training and accreditation in the intensive care: document for the World Interactive Network Focused on Critical Ultrasound (WINFOCUS) Cardiovasc Ultrasound 2008 6 49 10.1186/1476-7120-6-49 18837986 PMC2586628

[B5] Moore CL Copel JA Point-of-care ultrasonography N Engl J Med 2011 364 749 757 10.1056/NEJMra0909487 21345104

[B6] Atar S Feldman A Darawshe A Siegel RJ Rosenfeld T Utility and diagnostic accuracy of hand-carried ultrasound for emergency room evaluation of chest pain Am J Cardiol 2004 94 408 409 10.1016/j.amjcard.2004.04.052 15276122

[B7] Spevack DM Spevack DM Tunick PA Kronzon I Hand carried echocardiography in the critical care setting Echocardiography 2003 20 455 461 10.1046/j.1540-8175.2003.03083.x 12848868

[B8] Vignon P Frank MB Lesage J Mucke F Francois B Normand S Bonnivard M Clavel M Gastinne H Hand-held echocardiography with Doppler capability for the assessment of critically-ill patients: is it reliable? Intensive Care Med 2004 30 718 723 10.1007/s00134-003-2128-x 14722628

[B9] Labovitz AJ Noble VE Bierig M Goldstein SA Jones R Kort S Porter TR Spencer KT Tayal VS Wei K Focused cardiac ultrasound in the emergent setting: a consensus statement of the American Society of Echocardiography and American College of Emergency Physicians J Am Soc Echocardiogr 2010 23 1225 1230 10.1016/j.echo.2010.10.005 21111923

[B10] Mondillo S Giannotti G Innelli P Ballo PC Galderisi M Hand-held echocardiography: its use and usefulness Int J Cardiol 2006 111 1 5 10.1016/j.ijcard.2005.07.002 16087257

[B11] Seward JB Douglas PS Erbel R Kerber RE Kronzon I Rakowski H Sahn LD Sisk EJ Tajik AJ Wann S Hand-carried cardiac ultrasound (HCU) device: recommendations regarding new technology. A report from the Echocardiography Task Force on New Technology of the Nomenclature and Standards Committee of the American Society of Echocardiography J Am Soc Echocardiogr 2002 15 369 373 10.1067/mje.2002.123026 11944016

[B12] Egan M Ionescu A The pocket echocardiograph: a useful new tool? Eur J Echocardiogr 2008 9 721 725 10.1093/ejechocard/jen177 18579497

[B13] Manasia AR Nagaraj HM Kodali RB Croft LB Oropello JM Kohli-Seth R Leibowitz AB DelGiudice R Hufanda JF Benjamin E Goldman ME Feasibility and potential clinical utility of goal-directed transthoracic echocardiography performed by noncardiologist intensivists using a small hand-carried device (SonoHeart) in critically ill patients J Cardiothorac Vasc Anesth 2005 19 155 159 10.1053/j.jvca.2005.01.023 15868520

[B14] Scholten C Rosenhek R Binder T Zehetgruber M Maurer G Baumgartner H Hand-held miniaturized cardiac ultrasound instruments for rapid and effective bedside diagnosis and patient screening J Eval Clin Pract 2005 11 67 72 10.1111/j.1365-2753.2004.00506.x 15660539

[B15] Vignon P Chastagner C Francois B Martaille JF Normand S Bonnivard M Gastinne H Diagnostic ability of hand-held echocardiography in ventilated critically ill patients Crit Care 2003 7 R84 91 10.1186/cc2360 12974974 PMC270721

[B16] Amiel JB Grumann A Lheritier G Clavel M Francois B Pichon N Dugard A Marin B Vignon P Assessment of left ventricular ejection fraction using an ultrasonic stethoscope in critically ill patients Crit Care 2012 16 R29 10.1186/cc11198 22335818 PMC3396274

[B17] Cardim N Fernandez Golfin C Ferreira D Aubele A Toste J Cobos MA Carmelo V Nunes I Oliveira AG Zamorano J Usefulness of a new miniaturized echocardiographic system in outpatient cardiology consultations as an extension of physical examination J Am Soc Echocardiogr 2011 24 117 124 10.1016/j.echo.2010.09.017 21074362

[B18] Frederiksen CA Juhl-Olsen P Larsen UT Nielsen DG Eika B Sloth E New pocket echocardiography device is interchangeable with high-end portable system when performed by experienced examiners Acta Anaesthesiol Scand 2010 54 1217 1223 10.1111/j.1399-6576.2010.02320.x 21039344

[B19] Lafitte S Alimazighi N Reant P Dijos M Zaroui A Mignot A Lafitte M Pillois X Roudaut R DeMaria A Validation of the smallest pocket echoscopic device's diagnostic capabilities in heart investigation Ultrasound Med Biol 2011 37 798 804 10.1016/j.ultrasmedbio.2011.02.010 21458144

[B20] Reant P Dijos M Arsac F Mignot A Cadenaule F Aumiaux A Jimenez C Dufau M Prevost A Pillois X Fort P Roudaut R Lafitte S Validation of a new bedside echoscopic heart examination resulting in an improvement in echo-lab workflow Arch Cardiovasc Dis 2011 104 171 177 10.1016/j.acvd.2011.01.003 21497306

[B21] Lang RM Bierig M Devereux RB Flachskampf FA Foster E Pellikka PA Picard MH Roman MJ Seward J Shanewise J Solomon S Spencer KT St John Sutton M Stewart W Recommendations for chamber quantification Eur J Echocardiogr 2006 7 79 108 10.1016/j.euje.2005.12.014 16458610

[B22] Jurcut R Giusca S La Gerche A Vasile S Ginghina C Voigt JU The echocardiographic assessment of the right ventricle: what to do in 2010? Eur J Echocardiogr 2010 11 81 96 10.1093/ejechocard/jep234 20124362

[B23] Weyman A Principles and Practice of Echocardiography 1994 2 Philadelphia, PA: Lea & Febiger

[B24] Kircher BJ Himelman RB Schiller NB Noninvasive estimation of right atrial pressure from the inspiratory collapse of the inferior vena cava Am J Cardiol 1990 66 493 496 10.1016/0002-9149(90)90711-9 2386120

[B25] Bland JM Altman DG Statistical methods for assessing agreement between two methods of clinical measurement Lancet 1986 1 307 310 2868172

[B26] Cohen J A coefficient of agreement for nominal scales Educational and Psychological Measurement 1960 20 37 46 10.1177/001316446002000104

[B27] Landis JR Koch GG The measurement of observer agreement for categorical data Biometrics 1977 33 159 174 10.2307/2529310 843571

[B28] Prinz C Voigt JU Diagnostic accuracy of a hand-held ultrasound scanner in routine patients referred for echocardiography J Am Soc Echocardiogr 2011 24 111 116 10.1016/j.echo.2010.10.017 21126857

[B29] Kobal SL Tolstrup K Luo H Neuman Y Miyamoto T Mirocha J Naqvi TZ Siegel RJ Usefulness of a hand-carried cardiac ultrasound device to detect clinically significant valvular regurgitation in hospitalized patients Am J Cardiol 2004 93 1069 1072 10.1016/j.amjcard.2003.12.066 15081463

[B30] Galasko GI Henein M Direct access echocardiography Int J Cardiovasc Imaging 2006 22 29 31 10.1007/s10554-005-0721-5 16374530

[B31] Filly RA Is it time for the sonoscope? If so, then let's do it right! J Ultrasound Med 2003 22 323 325 12693615 10.7863/jum.2003.22.4.323

[B32] Roelandt JR Ultrasound stethoscopy: a renaissance of the physical examination? Heart 2003 89 971 973 10.1136/heart.89.9.971 12922991 PMC1767811

[B33] Galderisi M Santoro A Versiero M Lomoriello VS Esposito R Raia R Farina F Schiattarella PL Bonito M Olibet M de Simone G Improved cardiovascular diagnostic accuracy by pocket size imaging device in non-cardiologic outpatients: the NaUSiCa (Naples Ultrasound Stethoscope in Cardiology) study Cardiovasc Ultrasound 2010 8 51 10.1186/1476-7120-8-51 21110840 PMC3003628

[B34] Expert Round Table on Ultrasound in ICU International expert statement on training standards for critical care ultrasonography Intensive Care Med 2011 37 1077 1083 21614639 10.1007/s00134-011-2246-9

[B35] Sievers B Kirchberg S Franken U Puthenveettil BJ Bakan A Trappe HJ Visual estimation versus quantitative assessment of left ventricular ejection fraction: a comparison by cardiovascular magnetic resonance imaging Am Heart J 2005 150 737 742 10.1016/j.ahj.2004.11.017 16209976

